# Mechanism of Human Tooth Eruption: Review Article Including a New Theory for Future Studies on the Eruption Process

**DOI:** 10.1155/2014/341905

**Published:** 2014-02-12

**Authors:** Inger Kjær

**Affiliations:** Orthodontics Section, Department of Odontology, Faculty of Health and Medical Sciences, University of Copenhagen, 20 Nørre Allé, 2200 Copenhagen N, Denmark

## Abstract

Human eruption is a unique developmental process in the organism. The aetiology or the mechanism behind eruption has never been fully understood and the scientific literature in the field is extremely sparse. Human and animal tissues provide different possibilities for eruption analyses, briefly discussed in the introduction. Human studies, mainly clinical and radiological, have focused on normal eruption and gender differences. Why a tooth begins eruption and what enables it to move eruptively and later to end these eruptive movements is not known. Pathological eruption courses contribute to insight into the aetiology behind eruption. A new theory on the eruption mechanism is presented. Accordingly, the mechanism of eruption depends on the correlation between space in the eruption course, created by the crown follicle, eruption pressure triggered by innervation in the apical root membrane, and the ability of the periodontal ligament to adapt to eruptive movements. Animal studies and studies on normal and pathological eruption in humans can support and explain different aspects in the new theory. The eruption mechanism still needs elucidation and the paper recommends that future research on eruption keeps this new theory in mind. Understanding the aetiology of the eruption process is necessary for treating deviant eruption courses.

## 1. Introduction

Tooth eruption has been examined in both animal and human tissues. In animal tissue it is possible to perform cross-sectional studies on the eruption process with the surrounding periodontal membrane and bone, both histologically and immunohistochemically. This is not possible in human tissues. Meanwhile, the eruption process has been studied longitudinally in extensive studies in humans with focus on both normal and pathological conditions. Both clinical and radiological studies have been performed in humans and animals. All studies on tooth eruption conclude that the eruption process or the mechanism behind eruption is not fully understood.

## 2. Animal Experimental Eruption Studies 

Animal experimental studies have generally demonstrated that the tooth follicle plays a major role as the structure that forms the path for the crown through the overlying bone during the eruption process [1–3]. Thus, Marks and Cahill [1] examined in an experimental study the effect on eruption after surgical removal of different parts of the follicle. They found that removal of either the basal or coronal halves of the follicle prevented eruption. They also saw that the eruption pathway created by bone resorption did not develop after removal of the follicle. They concluded in their study [1] that resorption and alveolar bone formation occurring around an erupting tooth are regulated by adjacent parts after the dental follicle.

In another experimental study [2] Wise et al. injected a soluble form of dexamethasone in rats with the purpose of analyzing its effect on tooth eruption. They found that dexamethasone injections accelerated the eruption process in rat incisors but not in rat molars. As rat incisors erupt continuously whereas rat molars just like human teeth are teeth of limited eruption they concluded that cautions must be taken in conclusion from rat incisors to human teeth [2].

In a succeeding study, Wise et al. [3] found that downregulation of osteoprotegerin (OPG) which is needed for tooth eruption is mediated by colony-stimulating factor-1 (CSF-1) expressed in the dental follicle of erupting teeth [3].

Accordingly, several experimental studies have been performed that show how the follicle functions in the resorption process that is invoked in the bone tissue during eruption.

It has also been evaluated in experimental studies what influence the innervation has on tooth eruption. Thus, it was shown in experimental studies that eruption stopped when the nerve connection to the teeth was interrupted [4].

Fujiyama et al. [4] found that denervation performed experimentally led to dentoalveolar ankylosis with decreased width of the periodontal space. They suggested in their study that the Malassez epithelium may be involved in the maintenance of periodontal space and that sensory innervation might be indirectly associated with it [4].

The path finding process of the nervous tissue to the developing tooth primordium has been demonstrated [5, 6]. Kettunen et al. [6] demonstrated that Fgfr2b mediated epithelial-mesenchymal interaction coordinates tooth morphogenesis and also dental trigeminal axon patterning. Neuroendocrine cells have been demonstrated in the Malassez epithelium [7, 8].

Other animal experimental studies are the well-known experimental studies by Mina and Kollar [9] on the odontogenetic potential of the tissues during tooth formation. Mina and Kollar [9] separated epithelial and mesenchymal components by enzymatic digestion. They found that mandibular mesenchyme must interact with mandibular epithelium in order to have the competence to induce teeth in nonodontogenic epithelium. The studies have later been elaborated by Jussila and Thesleff [10] who showed how the controlling of the tooth formation in mice until the 12th foetal day is located in the epithelium after which the controlling of the continued tooth formation transfers to the ectomesenchyme. The studies also showed that the ectomesenchyme in, for example, molar regions is not the same as the ectomesenchyme in the incisor region. These early stages in the tooth formation should be kept in mind when the later developmental stages including eruption are to be elucidated.

## 3. Normal Tooth Eruption in Humans

Studies on human tooth eruption have examined several aspects of the eruption process. As opposed to the experimental studies, human studies are rarely histological, but often clinical and radiological studies.

*Eruption Times*. Eruption times have been studied clinically in the primary and permanent dentitions [11, 12]. The studies focus on the time when different teeth penetrate the overlying gingiva and appear in the mouth. The studies also show that eruption times are gender specific. Girls' teeth erupt sooner than boys' [11, 13, 14]. 

*Correlation between Eruption, Dental Maturity, Skeletal Maturity, and Age*. It has been examined how eruption times are coordinated with the osseous maturity of an individual and it has been shown that this correlation is important to consider. In the study by Svendsen and Björk [15] it is hypothesized that third molar impaction in the mandible is a consequence of late third molar maturation and early skeletal maturation. A strong correlation has been shown between eruption time and dental maturity. The teeth normally erupt when they have reached 2/3 root length [16]. Under pathological conditions, as for example, in Hyper IgE syndrome, the teeth even though they have reached full root length do not erupt at all [17, 18].

Furthermore, the individual correlation between chronological age and eruption time is weak [13, 14]. Thus, it appears that the increasing maturity of the teeth and also the eruption follow their own path. In that connection it is important to clarify that no scientific studies have shown which parameters affect the maturity of the teeth.

*Correlation between Eruption of Different Teeth*. In a larger population study on eruption times in 12,000 boys and 12,000 girls, Parner et al. [12] found a significant bilateral congruence between the eruption of the individual teeth. As an example the eruption times of the four permanent first molars are very closely correlated, while the eruption times of the first molars are not correlated with those of nonmolars. On the other hand, there is a clear correlation between the eruption times of molars within the molar fields [12]. It can thus be expected that a second permanent molar erupts early if the patient's first molar erupted early and there is thus a correlation between eruption times within tooth groups, but not between tooth groups.

It is normally stated that a child's first molar and lateral/central incisors erupt at the age of 6-7 years, but eruption in these different tooth groups is not interrelated. A child can have its first molars at the age of 6 years and the incisors several years later [12].

*Length of Eruption Process*. The eruption process in itself, or the moving of the tooth bud, begins with the early root formation [19]. The period from this early time and until the appearance of the teeth in the mouth is called the eruption time. There is great difference between the eruption times of different teeth and the time it takes for tooth to erupt is therefore different.

*Variations in Eruption Intensity*. Studies on individuals in pre-, during, and postpuberty have shown that a continuous eruption takes place after the teeth have reached occlusion [19–21]. These studies have been performed with metal indicators inserted in the jaws and are therefore useful for showing how the alveolar process grows in relation to tooth eruption. The studies [19–21] show that the two developmental processes, eruption and growth of the alveolar process, are processes that are mutually correlated. It has previously been shown that growth in body height is strongly correlated with jaw growth [19]. It has thus also been demonstrated that the growth of the alveolar process is weak in the prepubertal period when height growth is also weak, while growth of the alveolar process increases significantly during puberty. During this period eruption accelerates and later diminishes when growth in height and on the alveolar process ends. Recently, it has been questioned whether early so-called bone loss in Juvenile Periodontitis is not a loss of bone due to infection but a lower level of alveolar bone mesially to the first mandibular molar caused by arrest in alveolar bone growth [22].

## 4. Pathological Eruption Courses in Humans

Important studies on human tooth eruption have been based on descriptions of deviant or pathological eruption courses. When a health condition is known to affect the oral cavity either generally or locally and when it is observed how this condition affects the eruption (general or local manifestation) it is possible to understand the normal eruption step by step. 

*Premature and Delayed Eruption*. Very little literature exists on patients whose teeth erupt much earlier or later than normally. Thus, the causes behind these premature or delayed eruption deviations are not known and the literature is based on few case descriptions of eruption times in different medical conditions [23–30]. Cohen mentioned in his study [23] that prenatal eruption of tooth occurs in 1 of every 2000 births. The reported association between natal teeth and serious, rare inherited syndromes suggests that particular caution should be exercised in examination of newborns having prematurely erupted teeth. Whyte et al. [30] describe how different rare metabolic bone diseases influence tooth eruption.

Delayed or absent eruption of the permanent dentition has been observed in a rare case disposing enamel agenesis and renal symptoms [24, 31]. This observation is in agreement with the observation by Martelly-Júnior [28] of a patient with hypoplastic enamel, renal malformation associated with delayed tooth eruption.

An overview of cases reported with delayed tooth eruption is given by Suri et al. [29]. This overview concludes the need for a more in-depth understanding of the underlying pathophysiology of delayed tooth eruption.

Meanwhile, it is known that teeth erupt later in certain syndromes. The condition when teeth are formed in the jaw but do not erupt is rare. It has been described in very few cases and occurs, for example, in GAPO syndrome [32]. Gapo syndrome is probably an autosomal-recessive condition of growth retardation, alopecia, and optic atrophy [32]. The cause of this eruption deviation in Gapo syndrome is not known.

**Figure 1:**
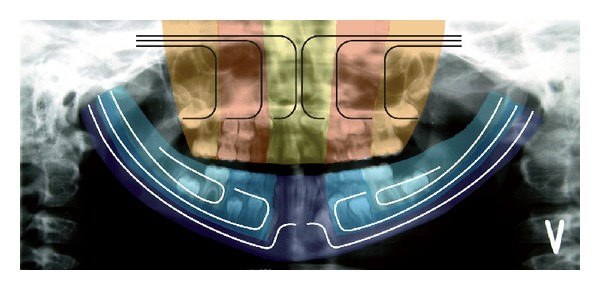
The embryological development of the jaws and teeth from the neural crest, shown schematically in colours on a panoramic radiograph. The different fields in the mandible are marked in blue. The white lines show three different courses of peripheral nerves to the teeth and bone in the mandible. In the maxilla, the bilateral frontonasal fields are marked in yellow, the bilateral maxillary fields in red, and the bimaxillary palatinal fields in orange. The black lines show three different courses of peripheral nerves to the teeth and bone in the maxilla.

**Figure 2:**
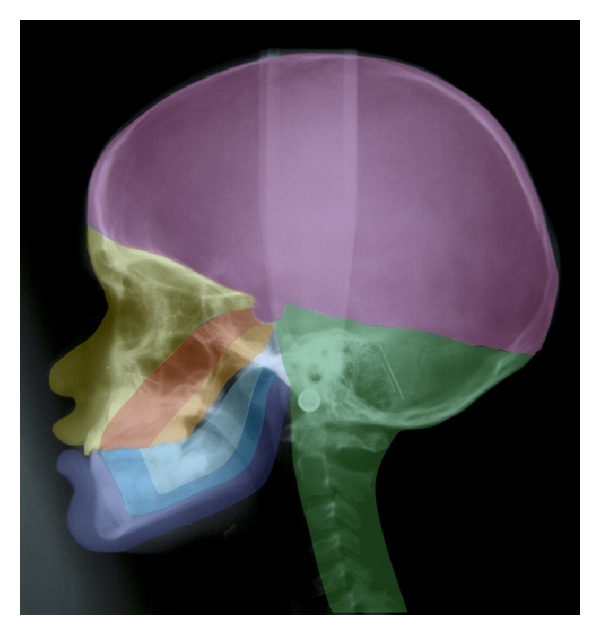
The embryological development of the cranium, jaws, and teeth from the neural crest and the notochord shown schematically in colours on a profile radiograph. The different fields in the mandible are marked in blue. The fields in the maxilla are marked in yellow (the frontonasal field), in red (the maxillary field), and in orange (the palatinal field). The theca cranii field is marked in purple and the occipital/cerebellar field in green [50].

**Figure 3:**
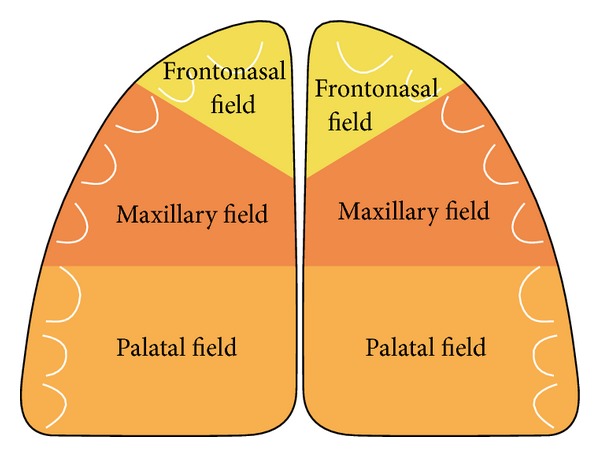
Schematic drawing of the palate. White semicircles mark the location of the maxillary teeth according to the coloured maxillary fields: yellow fields indicate frontonasal fields, red fields indicate maxillary fields, and orange fields indicate palatinal fields (see Figure 1).

*Local Eruption Deviations*. It is characteristic that eruption deviations can occur generally in dentitions as described in the above cases and also locally in single teeth or within tooth groups [31, 33–45]. Tumours have influenced normal eruption regionally [39, 40], and so have supernumerary teeth [35]. An example of an eruption deviation affecting canines and premolars is described in Hyper IgE syndrome where the primary canines and the primary molars are not shed, while the root formation of the underlying teeth continues to full length [17, 18, 38, 42]. The cause of regional eruption disturbances associated with alveolar bone malformation may be seen in the jaw fields that have been introduced during recent years based on prenatal studies on cranial and jaw development [46–50]. These fields are demonstrated in Figures 1–3 and explained later in the paper. Treatment of regional eruption disturbances is often interdisciplinary [51].

Eruption deviations also occur as isolated findings in single teeth. These deviations occur in, for example, the permanent first molar or the second molar [52–58]. Primary retention of molars occurs before eruption and the cause could be either space problems or failure in the dental follicle's ability to initiate resorption of the overlying bone [44, 45, 53, 54]. Secondary retention of permanent molars occurs after the molar has penetrated the gingival [53]. The aetiology is not fully understood. Raghoebar et al. [57] have found in a histological study of 26 secondarily retained molars interradicular ankylosis in 81% of the cases. It was concluded in that study that the ankylotic areas in several cases could not be detected radiographically [57]. Barberia-Leache et al. [55] have in an extensive study analyzed ectopic eruption of the maxillary first permanent molar [55]. Association between ectopic eruption of maxillary canines and first molars has also been reported [55]. Thus, 23% of 30 patients with maxillary canine ectopia had a diagnosis of ectopia early in childhood of the first maxillary molar. The explanation for this presumed association has not been given.

In the primary dentition eruption arrests are often seen in molars [59, 60]. Less severe infraposition of primary molars does not require treatment due to natural exfoliation [59]. More severe secondary retention of primary molars results in extraction [60] due to ankylosis. 

*Ectopic Eruption*. Every single permanent tooth can erupt ectopically. The prevalence of ectopic eruption is different for individual teeth.

Most common in this connection is ectopic eruption of the maxillary canines [36, 61–72]. The aetiology behind this ectopic condition is intensively discussed. Peck et al. [68] have focused on a genetic background for palatally displaced canines. Becker and Chaushu [69] have in an extended study compared dental ages in patients with bucally displaced canines with a control group with normally located canines. Approximately half the subjects with palatal displacement exhibited a late-developing dentition while the timing in dental development in the remaining subjects was normal [69]. Buccal displacement of maxillary canines was not associated with a retarded dental development but demonstrated dental development similar to conditions seen in the control group. This study supports the idea that there are different aetiologies for the occurrence of buccal versus palatine canine ectopia [69].

It has also been described in the literature that an association exists between palatally ectopic canines and small malformed and missing teeth in the dentition [62, 69, 70]. Sacerdoti and Baccetti [71] analyzed a sample of 5000 subjects and found that the prevalence rate of palatally displaced canines was 2.4% with a male to female ratio of 1 : 3 [71]. Skeletally they reported a reduced vertical relationship in patients with palatally displaced canines. When they in that study [71] compared unilateral palatally displaced canines with bilateral palatally displaced canine cases they found that unilateral displacement was associated with agenesis of upper lateral incisors whereas bilateral displacement was associated with third molar agenesis [71]. This is again a finding which has not been explained.

Concerning space in the maxillary dental arch, Jacoby [72] found that 85% of the palatally erupted canines have sufficient space in the maxillary dental arch for eruption. This was later confirmed by Artmann et al. [65]. Also deviations in the cranio-facio-skeleton have been reported in cases with ectopic maxillary canines [63, 64]. Resorption of maxillary lateral incisors due to ectopic eruption of maxillary canines is a severe clinical problem, which is in focus in the literature on ectopic maxillary canines [61].

Transposition, which is an eruption deviation characterized by the shifting of place in the dental arch of single teeth causing treatment problems, is also a well-known eruption deviation in the permanent dentition [73]. In these dentitions craniofacial alterations in the maxillary skeleton have also been reported [66]. This specific type of eruption deviation is seemingly not described in the primary dentition.

Ectopic eruption of other teeth such as mandibular canines and third molars [74, 75] is described in the permanent dentition. Transmigration of a mandibular canine is a rare condition with unknown aetiology [76, 77].

With regards to aetiology, speculations behind these ectopic eruption courses are many. Most often genetic conditions are defined as the cause of ectopia [68, 75, 78], but that is not always the case. Ectopia can also be caused by deviations in space that may be hereditary, just as seen in small jaws, but can also be acquired due to early tooth extraction or due to primary teeth that are not shed. Additionally, a correlation between morphological ectodermal deviations in dentitions and ectopia has been described [65]. The size, growth, and osseous maturity of the jaw are also parameters that play a role in the understanding of the aetiology behind ectopia [64]. The space condition and how to analyze space experimentally, especially for third mandibular molar eruption, have been in focus in several reports.

## 5. The Causes of Tooth Eruption or the Mechanism behind Tooth Eruption 

When all these eruption aspects are comprised they provide a good insight into *how* tooth eruption progresses, *when* the teeth erupt, and *where* they erupt, but we have no coherent understanding of *why* the teeth erupt. When we do not know the aetiology behind eruption and cannot explain the eruption mechanism, then we cannot perform aetiology-based treatment. We can attempt to guess a treatment as, for example, surgical exposure of a first permanent molar that has primarily arrested eruption, because we have experienced that this treatment encourages eruption, but we do not know whether it is the crown follicle or the overlying gingiva, the alveolar bone or perhaps other factors that cause the arrested eruption. Several reviews from experienced researchers have, like the one from Marks and Schroeder [78], discussed the mechanism of tooth eruption, which is still not understood. Marks and Schroeder conclude in their review from 1996 [78], entitled “Tooth eruption: theories and facts”, that “the mechanisms of tooth eruption (i.e., the answer to the question of how and why teeth erupt) have been a matter of long historical debate. This review focuses on human and other mammalian teeth with a time- and spacewise limited period of eruption and analyzes recent observations and experimental data on dogs, rats, primates, and humans in a framework of basic biological parameters to formulate a guiding theory of tooth eruption. Acknowledging basic parameters (i.e., that teeth move in three-dimensional space, erupt with varying speed, and arrive at a functional position that is inheritable) eliminates a number of previously held theories and favors those that accommodate basic parameters, such a alveolar bone remodelling in association with root elongation, with possible correction factors in the form of cementum apposition and periodontal ligament formation. We have critically analysed, summarized, and integrated recent findings associated with preeruptive movements of developing teeth, the intraosseous stage of premolar eruption in dogs, molar eruption in rodents, and premolar and molar eruption in primates. The variable speeds of eruption are particularly important. In conclusion, the basic principles of tooth eruption depend on the type of signals generated by the dental follicle proper, the conditions under which teeth are moved, and the clinical understanding to be derived from this knowledge.”

What is said in textbooks?

If we look at the explanation and causes presented in the textbooks for the eruption process, we cannot find a clear answer either. We can read that some authors suggest that the eruption force is connected with the force that occurs when the tooth root grows, that is, suggesting an association between eruption force and root extension [79]. In 1992, Avery thus writes in the textbook *Essentials of Oral Histology and Embryology* on page 80: “Of the numerous causes of tooth eruption, the most frequently cited are root growth and pulpal pressure. Other important causes are cell proliferation, increased vascularity, and increased bone formation around the teeth. Additional possible causative agents, which have been noted include: endocrine influence, vascular changes, and enzymatic degradation. Probably all these factors have an influencing role but not necessarily independently of each other. Although all the factors associated with tooth eruption are not yet known, elongation of the root and modification of the alveolar bone and periodontal ligament are thought the most important factors. These events are coupled with the changes overlying the tooth that produce the eruption pathway.”

A connection between pulpal and periodontal reactions has also been mentioned as a causal factor in eruption [79, 80]. Thus, Bath-Balogh and Fehrenbach write in their textbook from 2006 on pages 84 and 85: “How tooth eruption occurs is understood, but why can only be theorized. No one can certify what forces “push” teeth through the soft tissues or can identify the timing mechanism that coincides with these eruptions. Each theory for eruption presents a problem in its conception. Root growth, existence of a temporary ligament, vascular pressure, contractile collagen, and hormonal signals genetic targets all have been used to explain eruption.”

The dental follicle surrounding the tooth crown has also been described as a factor decisive for the eruption process. Koch and Paulsen state the question regarding eruption mechanism in their paediatric textbook from 2009 thus on page 198: “The eruption path is determined by genetic and local environmental factors. One of the most important local environmental factors is crowding among the developing and erupting teeth. Tooth eruption is a biological process, which is still not fully understood. The process is accompanied by multiple tissue changes, such as resorption and apposition of the alveolar bone, and development of the root and periodontium.”

The problem is still how the tooth is elevated in the jaw. In general it can be concluded that the individual eruption pattern is inherited, that is, genetic, and that this pattern is also affected by local and general external factors. Many major textbooks do not mention the aetiology behind eruption and some only state that it is unknown [79–82]. In the textbook *Oral Anatomy*, *Histology and Embryology* Berkowitz et al. [82] have the following comments on page 363 to the question on the mechanism behind tooth eruption: “Although no one theory to explain the generation of eruptive force(s) is yet supported by sufficient experimental evidence, the brief review that follows will show that the eruptive mechanism: (1) is a property of the periodontal ligament (or its precursor, the dental follicle); (2) does not require a tractional force pulling the tooth towards the mouth; (3) is multifactorial in the more that one agent makes important contributions to the overall eruptive force; and (4) could involve a combination of fibroblast activity (although the evidence to date remains poor) and vascular and/or tissue hydrostatic pressures.”

Furthermore, Berkowitz et al. write on page 363: “Having established that the connective tissues around the developing tooth are most likely to be the source of the eruptive mechanism, two major systems have been implicated in the generation of the eruptive force. One view holds that the force is produced by the activity of periodontal fibroblasts through their contractility and/or motility; the other vascular and/or tissue hydrostatic pressures in and around the tooth are responsible for eruption. Whatever the system implicated in the eruptive mechanism, the evidence should be judged according to the following five criteria.

1. The proposed system must be capable of producing a force under physiological conditions that is sufficient to move a tooth in a direction favouring eruption.
2. Experimentally induced changes to the system should cause predictable changes in eruption.
3. The system requires characteristics that enable it to sustain eruptive movements over long periods of time.
4. The biochemical characteristics of the system should be consistent with the production of an eruptive force.
5. The morphological features associated with the system should be consistent with the production of an eruptive force.”

What can be concluded from scientific literature?

Animal experimental studies support the theory that the follicle is of importance for the eruption process [2, 3, 83–85] and have also shown that innervation plays a specific role in tooth eruption [4, 86–90].

There is no doubt that the bone tissue surrounding the tooth and the general growth conditions in the body play a role [21, 91–97]. In conditions with abnormal bone such as that observed in osteopetrosis then tooth eruption is affected [95], but the bone quality is not the only factor, which can explain the eruption process.

Human studies have suggested that there is no convincing correlation between early tooth formation (before crown formation) evaluated radiographically and the innervation pattern of the jaws [98].

Even though much is known about several aspects in the human tooth eruption we cannot explain what it is that causes a tooth to move in the jaw after crown formation and gradually erupt, often in a very long eruption path (longer than the root of the tooth) to its final place in the tooth row.

Considering that the phenomenon of tooth eruption and specifically pathological tooth eruption plays a major role in both clinical and theoretical dentistry there is surprisingly sparse literature on the subject. This is no doubt due to methodological difficulties. Experimental studies on animal tissue cannot uncritically be transferred to human conditions. Meanwhile, the eruption process cannot be studied on a molecular level sufficiently and furthermore not longitudinally in human tissues because teeth have to be extracted, which separates the teeth from the periodontal membrane and the surrounding bone.

## 6. New Theory behind the Eruption Process

As we cannot understand what causes a tooth to erupt and at the same time claim that the eruption process cannot be studied on animal experimental material and uncritically transferred to humans and secondarily that also human material have its methodological limitations, how can we then form a hypothesis for the eruption process?

This proposed hypothesis must be formed based on experience from human material. It is logical to turn to experiences from pathological and genetic material and to analyse what are the consequences of different diseases for different tissue types and for the eruption process. And then try to gather the information from pathological eruption processes and create a hypothesis for the eruption mechanism. It is not easy, but still it is more difficult based on observations of normal eruption courses to understand, for example, why the permanent first molars erupt at the same time in all four quadrants. It can be registered, but it cannot be explained.

Another way to approach the eruption problem is to combine knowledge from histological and histochemical studies of human teeth and jaws from different time before birth with similar studies of teeth including periodontium after birth. Basic knowledge on tooth tissue and the tissue that surrounds teeth can thus be analyzed in normal foetuses and pathologically genetically deviant foetuses. When this knowledge on tissue and genetics is transferred to the postnatal dentition, a hypothesis can be proposed based on a scientific background. This latter method forms the basis for the presented theory.

### 6.1. Background for the Theory

Before we try to understand what initiates, moves, lifts, and forms the path for a tooth primordium during eruption it is necessary to look at the early embryonic jaw formation and tooth formation.

### 6.2. Early Jaw Formation

The mandible and maxilla are formed in the early embryonic developmental stage from neural crest cells. These cells migrate from different areas on the neural crest of the neural tube with different molecular-biological origin. The cells with the different origins migrate to the different regions, also known as fields, to the jaws [49]. These fields are schematically marked on a panoramic radiograph, seen in Figure 1. The fields are characterized by having a separate innervation and regionally specific ectomesoderm, which is shown in the regions in Figure 1.

Figure 2 demonstrates the molecular-biological fields in the cranium [49]. These fields are different not just in the cranium and jaws but also in the dental arches [49]. The fields have different molecular origins and different innervations. They are shown in the dental arch and palate in Figure 3.

### 6.3. Early Tooth Formation

**Figure 4:**
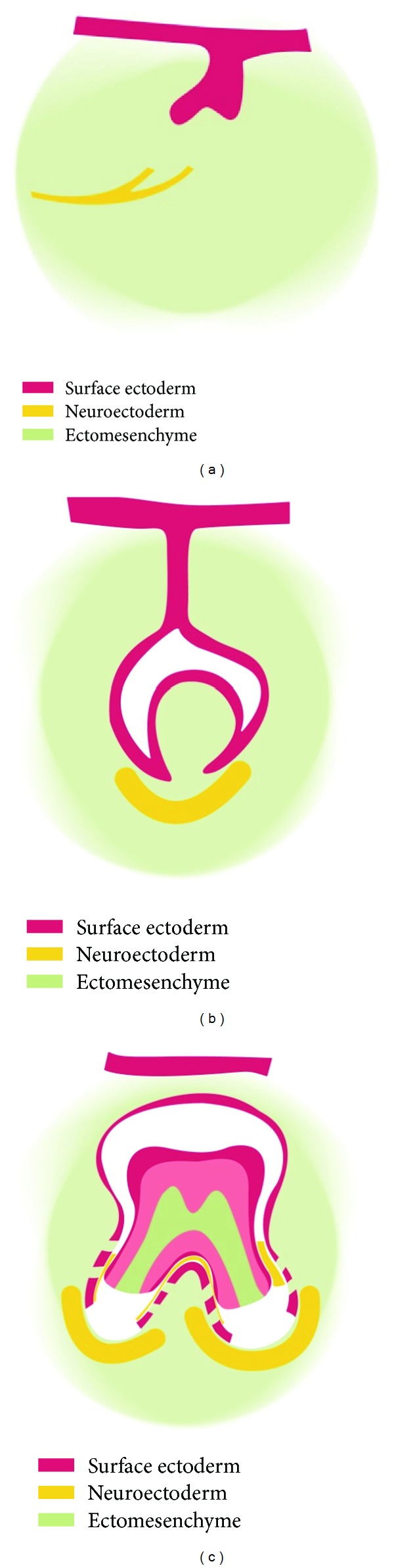
Schematic drawing of different stages ((a), (b), and (c)) in the formation of the human tooth primordium before eruption. Red indicates surface ectoderm/mucosa, green indicates ectomesenchyme, and yellow indicates peripheral innervation.

The early tooth formation is comprised of an ectodermal epithelial bud surrounded by regionally specific ectomesenchyme [48, 86, 91]. The nerve supply to the early tooth primordium, which is under rapid development, goes through a complicated path-finding process to the tooth primordium and gathers to begin with around the apical part of the primordium [91]. Quickly, the primordium develops through the well-known cap and bell stages. During these stages the innervation spreads and surrounds both the apical and the coronal parts of the primordium. Later, the reaction for nerve tissue is seen most strongly apically. The formation and tissue components in the early tooth formation are demonstrated in Figure 4. The innervation thus comprises a very important tissue component in the apical root sheet or root membrane that could also be designated the root follicle. There is no epithelium in the root follicle in contrast to the follicle around the tooth crown that has a pronounced inner layer of epithelium and only a light outer layer of innervation.

In summary, these are thus the tissue types: ectoderm, ectomesenchyme, and nerve tissue that are responsible for the early tooth formation.

### 6.4. Late Tooth Formation

**Figure 5:**
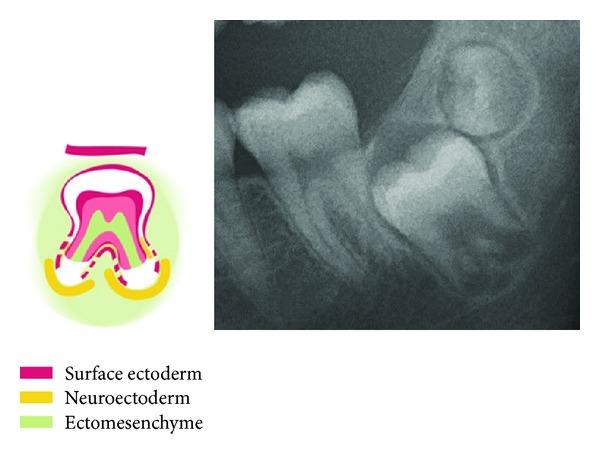
Schematic drawing and radiograph showing the morphology of a mandibular first molar shortly before eruption. Note the ectodermal cell layer of the follicle surrounding the crown and the strong innervation in the root sheet.

**Figure 6:**
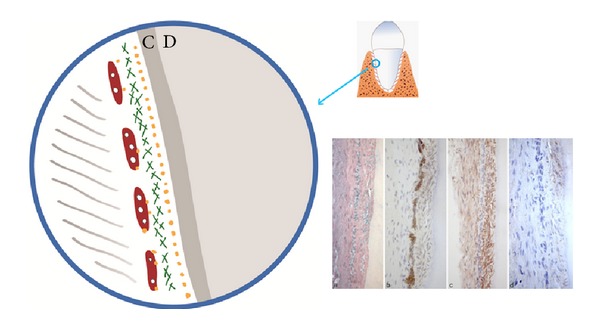
Schematic drawing of the cell layer in the root-close part of the periodontal membrane. D is root dentin and the cement layer C is marked on the surface. A layer of peripheral nerves (yellow dots) is seen the closest to the cement layer. Above is a dense fibre layer of ectomesenchymal origin (green lines) and outermost towards the more loose periodontal membrane the epithelial rests of Malassez are located (reddish-brown). This structured layer is called the peri-root sheet. Inserted in the figure are previously published histological sections showing the three cell layers [101].

The tissue types that influence the early tooth formation are the same tissue types that can be traced in the postnatal tooth formation (Figure 5). The crown follicle is comprised of an inner layer of ectoderm and an external layer of cell-dense ectomesenchyme. The periodontal membrane close to the root is called the peri-root sheet and is comprised of an inner nerve layer covered by a closely knit fibre layer of ectomesenchyme and outermost an ectodermal cell layer (Malassez cell layer) [92, 99, 100] (Figure 6). The apical root sheet or root membrane, suggested to be called the root follicle, is comprised of a strong layer of innervation and of a membrane-like layer of ectomesenchyme.

## 7. The New Theory of Eruption

*Hypothesis*. A tooth that will erupt depends on

1. space in the eruption path,
2. lift or pressure from below,
3. adaptability in the periodontal membrane.

Ad. 1. The crown follicle destroys overlying bone tissue and thus creates the necessary space in the eruption path. The molecular-biological processes depend on the ectoderm in the dental follicle and have been thoroughly described in animal experimental studies.

Ad. 2. The hypothesis is that the root membrane functions as a glandular membrane. The innervation in the membrane [91] causes, as in the glandular end-cells, an overpressure that supplants to the root surface, periodontal membrane, and pulp tissue [88, 102]. This pressure causes the tooth to elevate in the eruption direction. The process can be compared to the innervation of a gland that affects pressure conditions and provokes glandular secretion. From a physiological textbook (Miles et al., *Clinical Oral Physiology*, 2004) [102] on salivary glands the following sentence is extracted from page 22: “The secretion of saliva is regulated by the reflexes involving the autonomic nervous system. […] The release of neurotransmitters from autonomic nerve endings activates specific cell surface membrane receptors on the salivary gland tissue thereby determining the flow rate and composition of saliva.”

In their textbook [82] Berkowitz et al. also focus on the neuronal signalling of importance for salivary secretion. The following is cited from the textbook, page 261: “The sublingual gland and the minor salivary glands can spontaneously secrete saliva, but the bulk of the secretion is nerve mediated. The parotid and submandibular glands do not secrete saliva spontaneously and their secretion is entirely nerve-mediated.”

**Figure 7:**
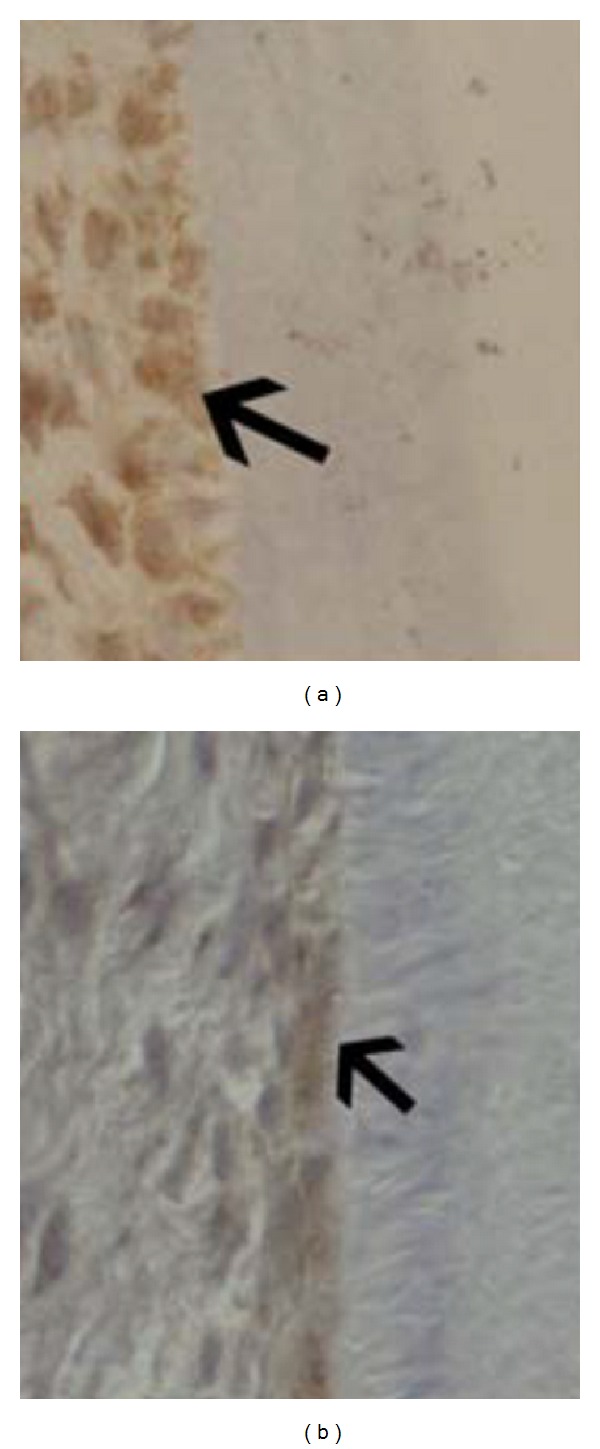
Immunohistochemical images of the root-close part of the periodontal membrane on a primary tooth from a child aged 6 (left) and a permanent tooth from a child aged 10 (right). The brown colour shows positive reaction for Caspase-3 which marks apoptosis activity [100]. The inner periodontal cell layer toward the root is thus under constant cell necrosis (restructuring) during the eruption process.

Ad. 3. The adaptability or reorganization in the periodontal membrane is the third factor that is essential for eruption. A proof for this reorganization process is that cell necrosis—apoptosis—has recently been demonstrated of the innermost root-close layer of the periodontium in erupting teeth.. An apoptotic cell layer has been demonstrated in both primary and permanent teeth undergoing eruption movements [100] (Figure 7).

*The Theory*. In summary, the aetiology behind the eruption process is that an innervation-provoked pressure in the apical part of the tooth results in an eruption that requires continuous adaptation from the periodontal membrane and the active movement of the crown follicle, destroying overlying bone tissue.

Conclusively, the membrane covering the apical part of the tooth root, the periodontal membrane, and the crown follicle are the three structures, which are involved in the eruption process. These three structures are interrelated and it is not unlikely that a pressure apically changes the periodontal membrane and at the same time triggers the crown follicle to resorption of the surrounding tissue.

## 8. Evaluation of the New Theory of Eruption

### 8.1. Normal Eruption Course

Parner et al.'s [12] study is specifically useful for testing the theory on normal eruption. Parner et al. documented that the eruption times are closely correlated within fields but not between fields. This suggests that the innervation, which is limited to the individual fields, or perhaps to the ectomesenchyme in the fields, influences the normal eruption.

### 8.2. Pathological Eruption Course

**Figure 8:**
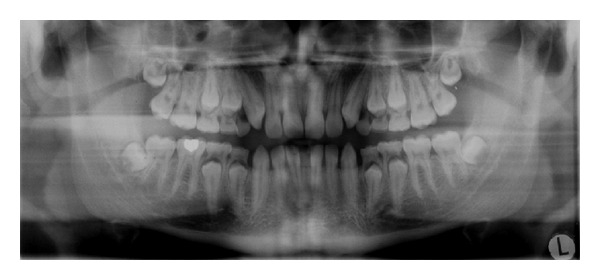
Panoramic radiograph of a 20-year-old male with the Hyper IgE condition. The figure shows deviations in the normal eruption pattern as premolars and two canines have fully formed roots but have not erupted.

*Peri-Root Sheet Defect and Arrested Eruption*. A defect in the peri-root sheet may be the cause of arrested eruption. A defect in the root sheet occurs if the peripheral nerves are destroyed by a virus function [49]. It must be presumed that virus destroys the myelin sheaths and that this prevents normal nerve function. An example of a virus infection that results in arrested eruption is mumps virus (Figure 8) [49, 51]. It appears that all teeth in the affected field can be arrested in eruption.

**Figure 9:**
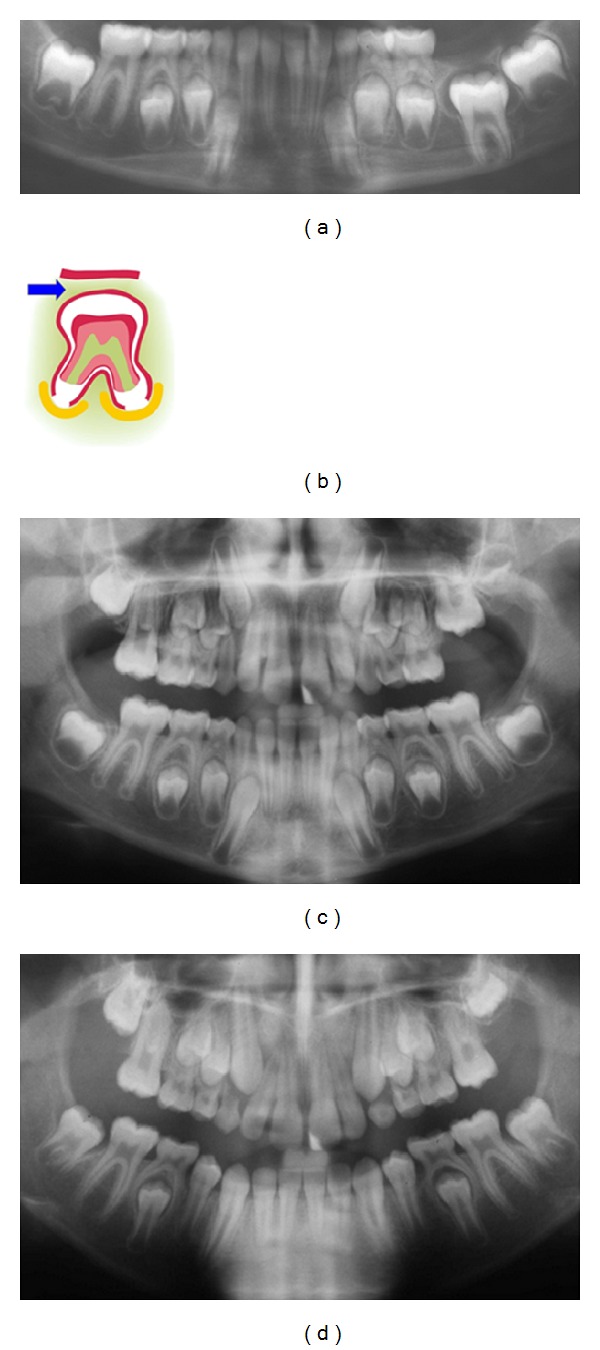
Drawing and radiograph showing a primarily retained permanent first molar. The cause is the crown follicle's lack of ability to destroy the overlying osseous tissue (blue arrow on the drawing). The upper radiograph shows primary retention of the left mandibular first molar from a 10-year-old boy. The bottom two panoramic radiographs from the same girl, aged 8 years and 8 months (left) and 10 years and 8 months (right). The two radiographs show that surgical exposure of the left maxillary first molar after the left radiograph was taken results in eruption of the tooth (right). At the same time, the radiographs show that the second maxillary molar in the field where the primarily retained tooth was located is delayed in formation. The condition in the left maxillary molar field has thus affected both eruption and formation of the teeth. The condition may be connected with a virus infection during the first years of the patient's life.

**Figure 10:**
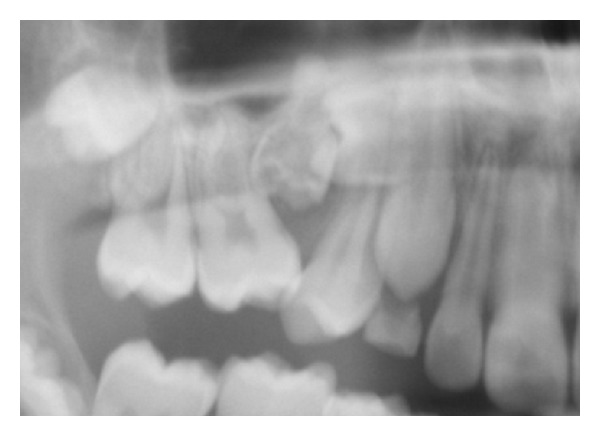
Section of a panoramic radiograph from a 14-year-old boy. The radiograph shows retention of the right primary second maxillary molar. The tooth has ankylosed and is not shed spontaneously. The tooth blocks normal development of the permanent dentition in the right side.

*Follicle Defect and Arrested Eruption*. If the epithelium of the crown follicle is inefficient and incapable of initiating resorption of the overlying hard tissue, eruption arrests (Figure 9). Resorption is normally initiated through molecular transport from the inner follicle to the outside. This can be seen in Hyper IgE syndrome where there is a general ectodermal insufficiency that, for example, also affects skin and lungs. Arrested or delayed eruption is also seen in certain types of ectodermal dysplasia [25, 104]. Arrested eruption of single teeth may also be caused by defects in the crown follicle. This condition is often seen in molars in the primary dentition and in first molars in the permanent dentition. The condition is called primary retention and may be treated by surgical exposure of the occlusal plane of the tooth. Shortly hereafter, the tooth normally erupts (Figure 10). What prevent the optimal function of the crown follicle is not known.

**Figure 11:**
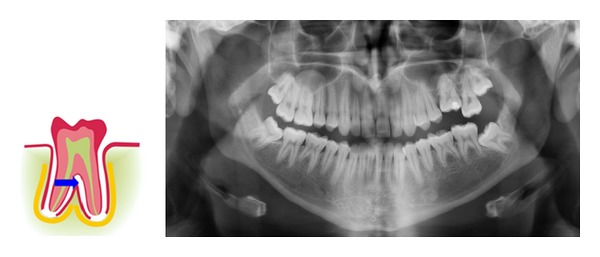
Drawing and panoramic radiograph (of a 20-year-old female) showing secondary retention of a permanent first molar in the left side of the maxilla. This first molar erupted normally, but ankylosed in the periodontal membrane after eruption. Therefore, the tooth could not continue the eruption course by “continued eruption” as other teeth do until growth ends after puberty. An arrow indicates that the process often begins interradicularly. The cause of the ankylosis is unknown.

**Figure 12:**
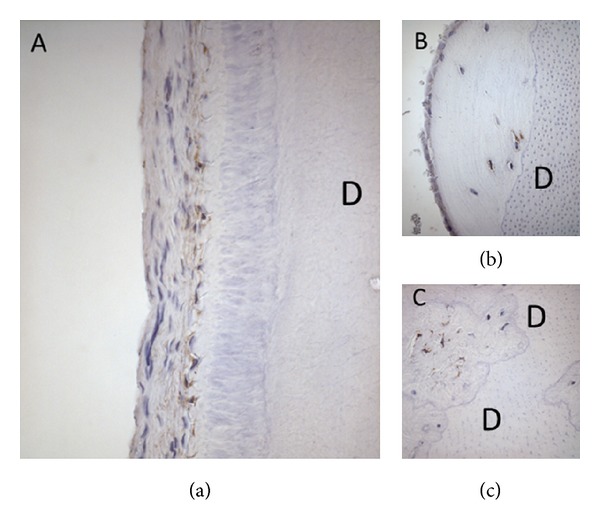
Three immunohistochemical sections of NeuN (nerve tissue markers) shown on otherwise uncoloured sections. D marks the dentin. Left: normally developed root surface (brown nerve cells) [89, 103]. Right: two sections of tooth root surfaces from teeth extracted due to arrested eruption. Upper: root dentin covered in hypercementosis-like tissue formations. Below: an unorganized pattern of dentin and bone tissue with nerve markings. It must be presumed that the very uneven surface dentin in the two images to the right is caused by resorption and that the resorption lacunae are later filled with tissue with osteogenic potential.

*The Periontal Membrane and Arrested Eruption*. If an inflammation occurs in the root-close periodontium, either due to trauma or due to other acquired disturbances in the periodontal membrane [101] a blood/lymphatic leakage occurs in the periodontal membrane. This accumulation of fluid may cause a pressure that results in resorption followed by a hard tissue deposit. This causes a decomposition of the normal structure of the periodontal membrane and the condition may result in ankylosis (Figure 11). If ankylosis or, for example, hypercementosis, occurs as demonstrated in Figure 12, the ability of the periodontal membrane to adapt naturally during eruption is weakened or interrupted. Which of the three tissue components in the root-close periodontal membrane that may have initiated arrested eruption is unknown, but it is likely that the inner nerve layer can play a role and that this contributes to arrested eruption within fields. Arrested eruption of single teeth after clinical eruption is designated secondary retention (Figure 11). In a case of secondarily arrested eruption the tooth is seemingly “lowered” into the jaw. The correct explanation for this phenomenon is meanwhile that the affected tooth due to ankylosis in the periodontal membrane does not follow the continuous eruption course and the continuous growth on the alveolar process as seen in the neighbouring teeth. The tooth stays where it was when the eruption obstacle in the tooth stopped the eruption process and did not follow the continuous eruption of the neighbouring teeth.

## 9. Conclusion

In summary, this spotlight paper demonstrates how seemingly very different conditions with eruption deviations can be explained based on existing literature on eruption presented with new knowledge on the development of the jaws and teeth before birth and by new insight into the tissue layers close to the root.

A new theory on the mechanism behind normal eruption is presented. It is shown by examples how pathological eruption courses can support the theory. The new theory on eruption forms a new basis for optimal treatment of eruption deviations.

What could be a future step in eruption research?

There is still much we do not know when it comes to eruption. A very important question is what controls the tooth maturation process and what initiates the eruptive movements of the tooth after crown formation. It must be presumed that these developmental factors are controlled endocrinologically.

Another one of many unanswered questions is how the connection occurs between the eruptively moving tooth and the increased growth of the alveolar process. Early studies have shown that bone cells are positive for nerve markers [92]. It could therefore be presumed that it is the nerve tissue that controls the growth of the alveolar process [92].

This paper is an input in the biological puzzle of tooth eruption with some new thoughts and ideas that may be included in future research.
